# Analysis of radon-associated squamous cell carcinomas of the lung for a*p53* gene hotspot mutation

**DOI:** 10.1054/bjoc.1999.0995

**Published:** 2000-02

**Authors:** Q Yang, H Wesch, K-M Mueller, H Bartsch, K Wegener, M Hollstein

**Affiliations:** Deutsches Krebsforschungszentrum (German Cancer Research Center) Im Neuenheimer Feld 280, Heidelberg, D69120, Germany; Institute of Pathology, Berufsgenossenschaftliche Kliniken Bergmannsheil, Universitaetsklinik, Buerkle-de-la-Camp-Platz 1, Bochum, D-44789, Germany; Institute of Pathology, Municipal Hospital of Ludwigshafen, Bremserstrasse 79, Ludwigshafen, D-67063, Germany

**Keywords:** p53, alpha-particles, uranium, mutation

## Abstract

Squamous cell lung carcinomas (SCC) from former employees of the Wismut uranium mining company (Saxony, Germany) were obtained from the Stollberg Archive in order to screen for p53 tumour suppressor gene codon 249 arg→met hotspot mutations, a putative molecular bio-dosimeter of alpha-particle (radon) exposure (Taylor et al (1994) *Lancet***343**: 86–87; McDonald et al (1995) *Cancer Epidemiol Biomarkers Prevent***4**: 791–793). Of the 29 archived samples of SCC meeting quality criteria for DNA analysis by polymerase chain reaction (PCR) and *Hae* III restriction enzyme digestion, two tumours were found that harboured this mutation. DNA sequencing confirmed the presence of a G to T base substitution within the *Hae* III site spanning codons 249 and 250 of the *p53* gene that results in replacement of arginine (wild-type) by methionine at residue 249. When these data are combined with those from our previous study of tumours from the Stollberg Archive in which 50 lung tumours were examined, (including nine SCCs), we conclude that the G→T (arg→met) codon 249 mutation prevalence in the Wismut miner cohort is not sharply elevated in lung cancers in general (two mutations/79 tumours), or specifically in SCCs of the lung (two mutations/38 SCC) when compared to data from lung cancer patients with no reported occupational exposure to radon gas. © 2000 Cancer Research Campaign


*British Journal of Cancer* (2000) **82**(4), 763–766
© 2000 Cancer Research Campaign
DOI: 10.1054/ bjoc.1999.0995, available online at http://www.idealibrary.com on IDEAL®

### Analysis of radon-associated squamous cell carcinomas of the lung for a *p53* gene hotspot mutation

**Q Yang[1], H Wesch[1], K-M Mueller[2], H Bartsch[1], K Wegener[3] and M Hollstein[1]**

[1]Deutsches Krebsforschungszentrum (German Cancer Research Center) Im Neuenheimer Feld 280, D69120 Heidelberg, Germany; [2]Institute of Pathology, Berufsgenossenschaftliche Kliniken Bergmannsheil, Universitaetsklinik, Buerkle-de-la-Camp-Platz 1, D-44789 Bochum, Germany; [3]Institute of Pathology, Municipal Hospital of Ludwigshafen, Bremserstrasse 79, D-67063 Ludwigshafen, Germany

**Summary** Squamous cell lung carcinomas (SCC) from former employees of the Wismut uranium mining company (Saxony, Germany) were obtained from the Stollberg Archive in order to screen for p53 tumour suppressor gene codon 249 arg→met hotspot mutations, a putative molecular bio-dosimeter of alpha-particle (radon) exposure (Taylor et al (1994) *Lancet* **343:** 86–87; McDonald et al (1995) *Cancer Epidemiol Biomarkers Prevent* **4:** 791–793). Of the 29 archived samples of SCC meeting quality criteria for DNA analysis by polymerase chain reaction (PCR) and *Hae*III restriction enzyme digestion, two tumours were found that harboured this mutation. DNA sequencing confirmed the presence of a G to T base substitution within the *Hae*III site spanning codons 249 and 250 of the *p53* gene that results in replacement of arginine (wild-type) by methionine at residue 249. When these data are combined with those from our previous study of tumours from the Stollberg Archive in which 50 lung tumours were examined, (including nine SCCs), we conclude that the G→T (arg→met) codon 249 mutation prevalence in the Wismut miner cohort is not sharply elevated in lung cancers in general (two mutations/79 tumours), or specifically in SCCs of the lung (two mutations/38 SCC) when compared to data from lung cancer patients with no reported occupational exposure to radon gas. © 2000 Cancer Research Campaign

**Keywords:** p53; alpha-particles; uranium; mutation

Sequencing of the p53 tumour suppressor gene in human tumours has provided several links between a specific kind of mutation and heavy exposure to a major risk factor. The association between sunlight exposure and tandem p53 mutations in skin cancer, and between tobacco smoke and transversions at specific p53 guanine hotspot residues in smokers' lung cancer, are two clear examples, whereas other examples of distinctive mutation profiles tentatively associated with specific risk factors await corroboration or plausible biological explanations.

In 1994 the presence of a specific mutation in the *p53* gene (codon 249 G→T, arg→met) in 31% of 52 lung cancers tested from uranium miners of the Colorado Plateau USA was reported (Taylor et al, 1994). The tumours examined were large cell cancers (LC) and squamous cell carcinomas (SCC) of the lung. The report was met with enthusiasm because the hotspot mutation might thus serve as a molecular bio-dosimeter of radon, but also with skepticism because alpha-particle radiation was not expected to cause such a discrete DNA base sequence alteration (Lo et al, 1993; Hei et al, 1994; Venitt and Biggs, 1994). Recent data, however, on oxidative damage induced by alpha-particle traversal exclusively through the cytoplasm of mammalian cells (Wu et al, 1999) raise the possibility that secondary effects of high energy particle radiation contribute to accumulation of G to T point mutations in DNA. A second study of the same Colorado Plateau mining cohort, but in which adenocarcinomas (ADC) rather than SCC and LC lung tumours were examined did not reveal the presence of the specific mutation in any of the 23 tumours analysed (McDonald et al, 1995), suggesting to investigators that there is histological tissue-type specificity for the arg→met codon 249 mutation.

Uranium miners from the Saxon ore district of former East Germany comprise the largest cohort of radon-exposed workers in the world (Spiethoff et al, 1997). These mines are now closed, but records confirm that working conditions were notoriously poor, resulting in high exposure to radon and mineral dust (Schuttmann, 1993). Tissue archives (hereafter referred to as the Stollberg Archive) containing tens of thousands of fixed tissue autopsy samples from employees of the Wismut mining company are being investigated now by pathologists and epidemiologists, and first results indicate that there is a high number of lung tumours in this Archive in comparison with pathology archives of non-mining areas in former East Germany. The first 50 lung tumours we screened from the Stollberg Archive revealed no arg→met hotspot mutations (Bartsch et al, 1995; Hollstein et al, 1997). In this initial study, nine of the 50 tumors were SCC. The aim of the present study, therefore, was to test a larger set of SCC from the Stollberg Archive for the putative hotspot mutation in order to explore the proposal (McDonald et al, 1995) that the codon 249 arg→met G to T mutation is an indicator, in SCC only, of high occupational radon exposure.

*Received 23 June 1999*
*Revised 4 October 1999*
*Accepted 6 October 1999*

*Correspondence to:* M Hollstein

### METHODS

### Sample preparation

Tumour blocks (formalin-fixed, paraffin-embedded material) of 123 lung cancer patients from the Stollberg Archive with histopathological diagnosis of SCC of the lung confirmed by two pathologists were sectioned and transferred to coated glass histology slides. Tumour cell-containing areas of haematoxylin and eosin-stained tissue sections were marked by the pathologists, and the percentage of tumour cells within the marked area was recorded for each case.

Of the 123 cases, 46 were chosen for this study that provided 30% or more primary tumour cells in a marked neoplastic area at least 16 $mm^2$ large. Unstained sections were dewaxed, and the tumour area was carefully microdissected and processed for DNA extraction as previously described (Hollstein et al, 1997). Buffer negative controls for use in subsequent polymerase chain reaction (PCR) experiments were prepared to monitor extraction reagents and procedures. All work up to the PCR amplification of genomic DNA was carried out in a special laboratory reserved for pre-PCR protocols. PCR set-up reactions were all performed with materials shielded behind plexiglass in a PCR Template Tamer™ (Oncor, Gaithersburg) hood equipped with a UV light source.

### Polymerase chain reaction

Reactions to amplify exon 7 of the p53 tumour suppressor gene were performed essentially as described previously (Bartsch et al, 1995). Reactions (50 μl) contained 2–4 μl of tumour DNA sample (an estimated 10–30 ng of DNA), and DNA was amplified with oligonucleotide primers specific for human *p53* gene intron sequences flanking exon 7. Hot-start DNA amplification was performed with an MJ Research Minicycler programmed at 95°C for 2 min; 35–45 cycles of 95°C (1 min), 60°C (1 min) and 72°C (30 s). Reaction products were visualized by electrophoresis of 5 μl PCR solution in 3.5% agarose gels (Sigma, Inc., Wide-Range (TM) agarose), staining for 5 min in 1 μg $ml^{-1}$ ethidium bromide solution, and photography on an UV illuminated table. Fragment size markers (*Phi*X-*Hae*III-digested DNA) were included in each electrophoresis.

### Analysis of PCR product for the presence of base pair substitutions at codon 249–250

PCR products were purified over Clontech-$H_2O$-DEPC columns (Clontech, CA, USA) and digested with restriction enzyme *Hae*III. Digestion reactions were as recommended by the manufacturer (Boehringer, Mannheim). Each DNA digestion reaction and corresponding DNA control incubation (no enzyme) were loaded side-by-side onto 3.5% agarose gels and electrophoresed at 90 V for 1 h. Gels were stained with ethidium bromide and photographed. The *Hae*III enzyme cleaves the wild-type p53 sequence at codon 249, but not a sequence with either the codon 249 AGG→ATG mutation or other mutants with a base change in the GG CC recognition site spanning codons 249–250. Uncleaved PCR product was taken as preliminary evidence for a mutation in codons 249–250, to be verified by repeat amplification and *Hae*III digestion of exon 7, and by direct DNA sequencing of (undigested) PCR product to identify the mutation. Codon 249 AGG to ATG mutations in tumours were then re-confirmed by preparing a new aliquot of genomic DNA extracted from a second paraffin tissue section from the tumour, amplification by PCR and DNA sequencing. Cycle sequencing reactions with BigDye™ (ABI, Applied Biosystems International, Weiterstadt, Germany) fluorescent dye-labelled dideoxynucleotide chain terminators were then performed according to manufacturer's specifications and analysed with an ABI 310 Genetic Analyzer. Samples were sequenced twice (5′→3′ and 3′→5 direction).

### RESULTS

Of the 46 SCCs selected for analysis of the *p53* gene, 29 (63%) yielded DNA of sufficient quality and amount to permit single-step PCR amplification and analysis of product by *Hae*III digestion. This percentage is not surprising considering that tissues from the Stollberg Archive were subjected to extensive (several weeks) formalin fixation, which is known to compromise severely the quality of DNA as template for PCR. The average number of years that these 29 patients with lung SCC were employed by the Wismut Mining Company was 20.2, with an average of 15.7 years underground activity. Most of these individuals began their work in the uranium mines between 1947 and 1951, when dry drilling procedures were still in practice, leading to very high radon exposure levels (Schuttmann, 1993, and unpublished Stollberg Archive records).

Two tumours (27 and 29) showed an altered *Hae*III enzymatic digestion profile and an AGG to ATG mutation in codon 249 (Figure 1). Repeat extraction of genomic DNA from new tissue sections of these tumours, and mutation analysis confirmed these results. The electropherogram of the DNA sequencing reaction shown in the upper panel of Figure 1A shows a hemizygous A**G**G to A**T**G mutation at codon 249 in tumour 29 (3′→5′ sequencing presents this mutation as a C to A substitution), whereas tumour 27 harboured a heterozygous A**G**G to A**T**G (arginine→methionine) mutation at the same site (Figure 1B upper panel, 5′→3′ sequencing). The normal p53 sequence at this location of the gene is displayed in the lower panels of Figure 1 for comparison. These sequencing data corroborate the *Hae*III digestion patterns shown in Figure 2. Thus we found two tumours in the set of 29 SCCs with the codon 249 arg→met mutation reportedly linked to radon exposure.

### DISCUSSION

With the data obtained from the work described here combined with results of our previous study, a total of 38 SCCs of the lung from the Stollberg Archive have now been screened, two of which were found to harbour the codon 249 arg→met mutation proposed to be a tumour biomarker for radon exposure. Twelve such mutations among the 41 SCCs examined from patients who worked in the uranium mines of the Colorado Plateau, USA were discovered by Taylor and colleagues (Taylor et al, 1994; McDonald et al, 1995). A comparison of results in SCC from the two cohorts (Colorado Plateau USA vs Stollberg Archive uranium miners) shows a clear difference in frequency ($P < 0.01$, $\chi^2$ statistic, Table 1). In p53-mutant lung tumours (all histological subtypes combined) from patients *not* known to have been exposed to radon gas occupationally, the codon 249 arg→met mutation is found occasionally, that is, in approximately one tumour per 150 tumours with p53 mutations (IARC Human Somatic Mutation Database, Hainaut et al, 1998). In the Stollberg Archive we found a prevalence of two per 79 lung tumours (histological subtypes combined) (Table 1), whereas 16 arg→met codon 249 mutations were found in a total of 75 lung tumours from the Colorado Plateau ($P <$ 0.001). It appears, therefore, that the arg→met codon 249 G to T

© 2000 Cancer Research Campaign

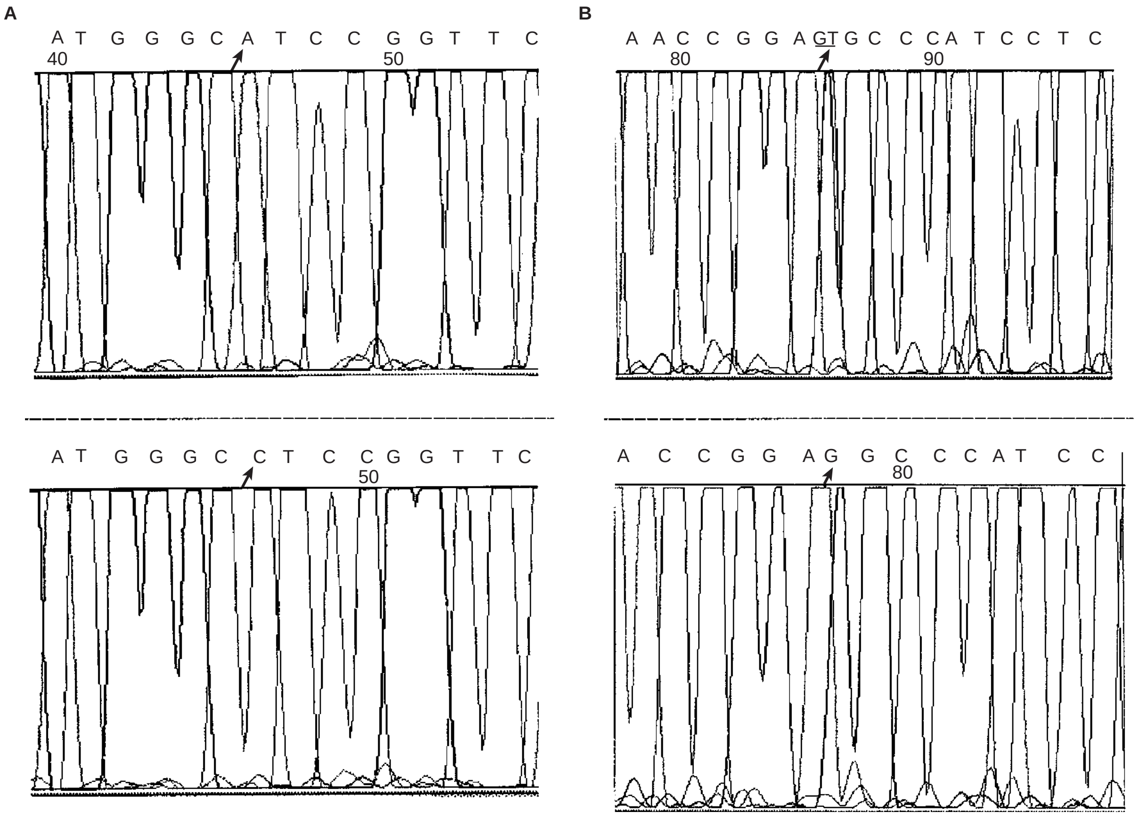

**Figure 1** DNA sequencing electropherograms, p53 exon 7. Upper panel of **A** shows a hemizygous codon 249 G:C to T:A mutation in tumour 29 (sequencing performed 3′→5′; mutation appears as a C to A base substitution), and upper panel of **B** presents the heterozygous codon 249 G:C to T:A mutation in tumour 27 (sequencing performed 5′→3′; mutation appears as a G to T base substitution, with both G and T signals at the mutation site). Lower panels show normal DNA sequence of p53 at this location for comparison

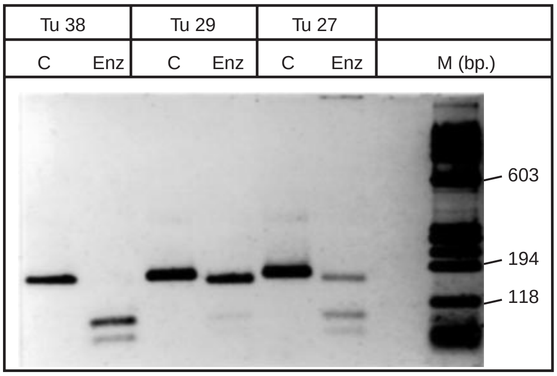

**Figure 2** RFLP analysis of p53 exon 7 PCR products. Agarose gel electrophoresis of PCR products from amplification reactions with DNA from three tumours is shown. Abbreviations: Tu, tumour; C, control PCR sample (no enzymatic digestion); Enz, enzymatic digestion of PCR product with *Hae*III restriction enzyme; M, Biosizer V (MBI) DNA fragment size markers. In lane 4 PCR product from tumour 29 remains undigested (mutant), whereas tumour 27 shows partial digestion (lane 6), suggesting the presence of a heterzygous mutation at the enzyme recognition site

**Table 1** Comparison of codon 249 arg→met hotspot mutation prevalence in archived lung cancers from two uranium mining sites

| Tumour type | Mining site | |
|---|---|---|
| | Colorado Plateau USA[a] | Saxony (Stollberg) Germany[b] |
| | Tumours with the mutation/ tumours tested | Tumours with the mutation/ tumours tested |
| Adenocarcinoma | 0/23 | 0/15 |
| Small cell lung cancer | 0/0 | 0/21 |
| Squamous cell carcinoma | 12/41 | 2/38* |
| Large cell carcinoma | 4/11 | 0/0 |
| Mixed or unclassified | 0/0 | 0/5 |
| | 16/75 | 2/79** |

[a]Data from Taylor et al (1994) and McDonald et al (1995). [b]Data from Bartsch et al (1995) and this report. $*\chi^2 = 7.79$; $P < 0.01$. $**\chi^2 = 13.17$; $P \ll 0.001$.

mutation is not a conspicuous marker of radon exposure in SCC lung cancer patients who were former uranium miners in the Saxon Ore district of Germany, nor in the miners with other histological subtypes of lung cancer. It has been suggested that this mutation was exceptionally prevalent in the cohort from the Colorado Plateau because of a second mutagenic exposure (e.g. a mycotoxin) specific to uranium mines in that area and therefore may not be a marker of radon exposure in Colorado Plateau workers either (Venitt and Biggs, 1994), but working circumstances that prevailed decades ago are difficult to verify now. With a larger database of p53 mutations in lung carcinoma in which details on histological subtype and patient exposure history are available, it would be possible to explore the still tenable hypothesis that the arg→met substitution at p53 residue 249, perhaps

© 2000 Cancer Research Campaign induced by oxidative damage from cigarette smoke and/or cytoplasmic damage from alpha-particle traversal, is preferentially selected for in bronchial squamous epithelium.

### ACKNOWLEDGEMENTS

This work was funded by the German Federal Ministry of the Environment under grant no. St.Sch.4057. The authors are grateful to K-M Muehlbauer for valuable technical assistance.

### REFERENCES

Bartsch H, Hollstein M, Mustonen R, Schmidt J, Spiethoff A, Wesch H, Wiethege T, Mueller K-M (1995) Screening for putative radon-specific p53 mutation hotspot in German uranium miners. *Lancet* **346**: 121

Hainaut P, Hernandez T, Robinson A, Rodriguez-Tome P, Flores T, Hollstein M, Harris CC, Montesano R (1998) IARC database of p53 gene mutations in human tumours and cell lines: updated compilation, revised formats and new visualization tools. *Nucleic Acids Res* **26**: 205–213

Hei TK, Bedford J and Waldren CA (1994) Mutation hotspot in radon-associated lung cancer. *Lancet* **343**: 1158

Hollstein M, Rice K, Greenblatt MS, Soussi T, Fuchs R, Sorlie T, Hovig E, Smith-Sorensen B, Montesano R, Harris CC (1994) Database of p53 gene somatic mutations in human tumours and cell lines. *Nucleic Acids Res* **22**: 3551–3555

Hollstein MC, Bartsch H, Wesch H, Kure EH, Mustonen R, Muehlbauer K-R, Spiethoff A, Wegener K, Wiethege T, Mueller, K-M (1997) *p53* gene mutation analysis in tumours of patients exposed to a-particles. *Carcinogenesis* **18**: 511–516

Lo YMD, Darby S, Noakes L, Whitley E, Silcocks PBS, Fleming KA and Bell JI (1995) Screening for codon 249 p53 mutation in lung cancer associated with domestic radon exposure. *Lancet* **345**: 60

McDonald JW, Taylor JA, Watson MA, Saccomanno G and Devereux TR (1995) p53 and K-ras in radon-associated lung adenocarcinoma. *Cancer Epidemiol Biomarkers Prevent* **4**: 791–793

Schuttmann W (1993) Schneeberg lung disease and uranium mining in the Saxon ore mountains (Erzgebirge). *Am J Ind Med* **23**: 355–368

Sidransky D and Hollstein M (1996) Clinical implications of the *p53* gene. *Annu Rev Med* **47**: 285–301

Spiethoff A, Wiethege T, Hollstein M and Wesch H (1997) German uranium miners studies: evaluation of the central archive of the Institute of Pathology at Stollberg: first results. *Appl Occup Environ Hyg* **12**: 964–969

Taylor JA, Watson MA, Devereux TR, Michels RY, Saccomanno G, Anderson M (1994) p53 mutation hotspot in radon associated lung cancer. *Lancet* **343**: 86–87

Vahakangas K, Samet JM, Metcalf RA, Welsh JA, Bennett WP, Lane DP and Harris CC (1992) Mutations of *p53* and *ras* genes in radon-associated lung cancer from uranium miners. *Lancet* **339**: 576–579

Venitt S and Biggs PJ (1994) Radon, mycotoxins, p53, and uranium mining. *Lancet* **343**: 795

Wu LJ, Randers-Pehrson G, Xu A, Waldren CA, Geard CR, Yu Z and Hei TK (1999) Targeted cytoplasmic irradiation with alpha particles induces mutations in mammalian cells. *Proc Natl Acad Sci USA* **96**: 4959–4964

*© 2000 Cancer Research Campaign*
